# Editorial: Genetic diversity and genomic insights into physiological adaptations of grasses to diverse ecological niches

**DOI:** 10.3389/fpls.2023.1143178

**Published:** 2023-03-07

**Authors:** Devendra Ram Malaviya, Ajoy Kumar Roy

**Affiliations:** Indian Grassland and Fodder Research Institute (ICAR), Jhānsi, India

**Keywords:** grass, physiology, abiotic stress, genetic diversity, ecological niches modelling

With approximately 12,000 species and 771 genera, Poaceae is one of the largest plant families, composed of many cereal crops and a large number of grass genera. Following their probable origin in the Late Cretaceous period, the grasses now occupy a wide range of habitats and are integral to the vast grasslands and rangelands spread over 26% of the Earth’s surface, supporting the livelihoods of more than 800 million people. These anthropogenic grasslands harbor a rich biodiversity in varied climates, ranging from tropical to temperate, and play a significant role in providing fodder for livestock and habitats for wildlife, checking soil erosion, supporting pollinators, and conserving about 20% of the world’s soil carbon stocks. The invitation to submit a discussion of both grasses and grassland ecophysiology was attractive because of their crucial role in ecosystems on the Earth.

These grasses can be seen growing at high temperatures (up to 45°C) down to sub-zero, at high salinity and alkalinity levels, at high moisture levels, and in areas with light stress and poor soil nutrients. Over the period of domestication and adaptation, these grasses acquired physiological properties in order to survive and developed tremendous phenotypic plasticity for adaptation to new niches. The C_4_ photosynthetic pathway makes them more competitive than C_3_ plants in open habitats with high light intensity and warm temperatures. Inter and intraspecies diversity for various physiological activities played a significant role in their adaptation. Grasses are environmentally compatible to different abiotic stress conditions through the development of complex adaptive mechanisms such as physiological, biochemical, molecular, and cellular changes. Hence, these grasses are likely to harbor genes for adaptation to diverse ecological niches and also abiotic and biotic stress tolerance genes. Additionally, they may also harbor genes for apomixis. Thus, in time, these genes may play a crucial role in crop breeding.

The genetic diversity for adaptation to such stresses and the genetic mechanism behind it are important knowledge resources for the future, in order to impart physiological adaptation on many crop species. The concerted efforts in this direction will require the study of various aspects: the diverse grass genotypes adapted to various stress conditions; intraspecies diversity vis-à-vis the physiological adaptation of grasses; the physiological mechanism of tolerance to abiotic stresses; the water-use efficiency of grasses; identifying genes and markers for stress tolerance; identifying genes for apomixis; and the evolution of photosynthetic pathways to survive under high light intensity and warm temperatures. Access to this type of comprehensive information will lead to improving abiotic stress tolerance in grasses as well as many crop species.

A total of six articles were published after rigorous screening. Two papers dealt with mineral and heavy metal stress conditions, and one was devoted to shade tolerance correlated with polyploidization. Two papers were related to genetic diversity analysis among populations of special ecotype rice landraces and wild rye. Both the papers advocated the proactive role of governments and other agencies to protect them. The success of invasive species was attributed to seed positions in a spikelet in another paper.

Fly ash, a byproduct generated by coal-based thermal power projects, is emerging as a significant environmental issue and requires a bioremedial solution. Hence, Kumar and Babu’s study, which explores suitable species for fly ash dumps, is relevant and important. They evaluated four native perennial grasses, namely, *Imperata cylindrica, Cenchrus ciliaris, Sporobolus diander*, and *Cynodon dactylon*, and recommended that all four species can be used to mitigate environmental contamination. Of the four species selected, except for *Imperata*, the other three are used as fodder and for amelioration of degraded land. The vertical penetration of roots was found to occur more in stressed conditions than in native soil. Thus, higher plasticity in the root biomass is considered an adaptation strategy of the plants in the stressed environment.

As discussed above, grasses harbor genes for many types of abiotic stress tolerance, and the advanced molecular tools enable the identification of the genes responsible and their further transferal to crop species. In this context, the study by Nie et al. is of great significance. It reports that abiotic stress-responsive NAC-type transcription factors play important roles in improving the stress tolerance of *Miscanthus sinensis*, a rhizomatous C_4_ perennial grass that is widely used for forage, bioenergy, and papermaking, etc. The study aimed to understand how abiotic stress response and signaling has contributed to the evolution of plant development. A total of 261 NAC genes were identified, which were distributed unevenly on 19 chromosomes. The expression patterns of 14 genes from the *M. sinensis* SNAC subgroup were analyzed under high salinity, PEG, and heavy metal conditions, and multiple NAC genes could be induced by the treatment.

Increasing biomass production in shaded conditions is a challenge. Hence, the work of Cao et al. to identify suitable shade-tolerant genotypes in Bermuda grass (*Cynodon dactylon*) is an important contribution. Of the three triploid hybrids evaluated (between the common Bermuda grass with diploid African Bermuda grass (*Cynodon transvaalensis*) and one common tetraploid cultivar of Bermuda grass), the common tetraploid variety Chunaxi showed excellent shade tolerance; this was followed by the triploid hybrid Tifsport, as compared to the other triploids Tifdwarf and Tifway. Better shade adaptation was linked to higher photosynthetic performance (Fv/Fm), chlorophyll content, gas exchange, and WUE in leaves, as well as better antioxidant capability. They inferred that during evolution, polyploidization could be beneficial for plants to develop better adaptation to various environmental stresses.

A large number of rice landraces have been reported from different parts of the world. The rice genome also has many conserved gene sequences. Liu et al. studied the genetic diversity pattern in 1481 germplasms of a special ecotypic rice landrace (Kam Sweet Rice—KSR) in China using SSR and SNP haplotype analysis. The study indicates that KSR is a special rice ecotype and a single rice ecotype, as well as a rich source of resistance to bacterial blight (Xa23) and rice blast (pid3). They also highlighted the role of the traditional culture of the local Dong people, who protect the landrace and its suitability to the organic ecological planting mode of the ‘rice–fish–duck’ symbiotic system.

Moreover, Xiong et al. reported that the Qinghai–Tibet Plateau (QTP) region is the diversity center of wild rye *Elymussibricus*, based on genetic variation studies in 23 natural populations in China using chloroplast DNA sequences and cpSSR markers. They cautioned that measures must be undertaken to deal with its intolerance to high temperatures, particularly continued exposure to warmer temperatures.

Wang et al. attributed the successful invasive property of *Aegilops tauschii* to its different positions in a spikelet, which result in plenty of flexibility at all stages of the seed, seedling, and growth/reproduction of the life cycle. They concluded that morphological and physiological variation between seeds in different positions, such as basal, middle, and distal, in a spikelet have the potential to spread germination over a period of time.

It is necessary to emphasize once again that more efforts are needed to look into the value of grass species not only as fodder and fuel resources, but also as a source of genes for many types of abiotic stress tolerance and apomixis.

## Author contributions

All authors listed have made a substantial, direct, and intellectual contribution to the work and approved it for publication.

